# Fetal and neonatal demise in zoonotic diseases: pathology and pathogenesis

**DOI:** 10.1128/iai.00242-26

**Published:** 2026-06-15

**Authors:** Laice Alves da Silva, Thaynara Parente de Carvalho, Monique Ferreira Silva Souza, Tatiane Alves da Paixão, Renée M. Tsolis, Renato Lima Santos

**Affiliations:** 1 Escola de Veterinária, Universidade Federal de Minas Gerais, Belo Horizonte, Brazil; 2 Instituto de Ciências Biológicas, Universidade Federal de Minas Gerais, Belo Horizonte, Brazil; 3 Department of Medical Microbiology and Immunology, University of California—Davis, Davis, California, USA; University of California Merced, Merced, California, USA

**Keywords:** zoonosis, fetus, mortality, *Brucella *spp, *Listeria monocytogenes*, *Toxoplasma gondii*

## Abstract

Zoonotic infections constitute a major cause of reproductive failure and perinatal mortality in humans and animals. Among the most relevant etiological agents are *Listeria monocytogenes*, *Brucella* spp., and *Toxoplasma gondii*, which, despite distinct biological properties, share the capacity to breach placental defenses, establish intracellular niches, and impair fetal development. This review aims to synthesize current knowledge on the pathological and pathogenic mechanisms underlying fetal and neonatal death associated with these pathogens, with a focus on placental infection, vertical transmission, and tissue injury. The outcome of infection is highly dependent on the gestational stage at exposure: early gestational infection is frequently associated with embryonic loss or resorption, whereas infection in later stages more commonly results in abortion, stillbirth, or congenital disease. Elucidation of these mechanisms is essential for the development of targeted preventive and control strategies for zoonotic reproductive diseases.

## INTRODUCTION

Zoonotic infections are a major cause of reproductive failure and perinatal mortality in both humans and animals, impacting public health and livestock production (1). Although *Listeria monocytogenes*, *Brucella* spp., and *Toxoplasma gondii* are not the only pathogens associated with reproductive losses, they have been extensively studied and are consistently linked to fetal pathology across multiple species. Despite their distinct features, these pathogens share a common ability to breach the placental barrier and disrupt fetal development, leading to abortion, stillbirth, or congenital disease. While these pathogens employ strategies of intracellular persistence and immune evasion, they utilize distinct molecular and cellular mechanisms to invade placental tissues and drive reproductive failure (2). Therefore, *L. monocytogenes*, *Brucella* spp., and *T. gondii* were elected as interesting models for gram-positive bacterium, gram-negative bacterium, and a protozoan in the context of this review since they are important drivers of negative reproductive outcomes.

The impact of these infections during pregnancy, as well as for embryonic or fetal development, varies depending on the gestational stage. During early pregnancy, infections can disrupt implantation and embryonic organogenesis, leading to embryonic mortality or resorption. As gestation progresses, microbial colonization of the placenta and fetus may trigger inflammatory and necrotizing lesions, vascular disruption, and placental insufficiency, culminating in abortion, stillbirth, or premature delivery. In the perinatal period, systemic dissemination of infection may result in neonatal sepsis, encephalitis, or respiratory failure, frequently contributing to perinatal mortality or chronic developmental deficits among survivors (3).

*Listeria monocytogenes* is a gram-positive facultative intracellular bacterium and a major cause of abortion and neonatal septicemia in humans and ruminants, whose trophoblast tropism enables placental crossing and fetal infection (4). *Brucella* spp. are gram-negative bacteria with strong placental tropism, causing necrotizing placentitis and fetal loss (5, 6). *T. gondii*, an obligate intracellular protozoan, causes congenital toxoplasmosis via transplacental transmission, potentially causing fetal death or malformations (7).

These pathogens illustrate the complex interplay between pathogen virulence mechanisms, placental morphophysiology, and the host immune response, which will ultimately determine pregnancy outcome during the course of infection. Although *L. monocytogenes*, *Brucella* spp., and *T. gondii* are well-recognized causes of reproductive losses, there are gaps in the literature, particularly in regard to fetal and neonatal pathology and pathogenesis. Here, we review the available data on fetal and neonatal pathology and pathogenesis during the course of infections with these pathogens, which may eventually contribute to improved diagnosis, prevention, and management of these zoonotic reproductive diseases.

## ROUTES FOR FETAL-EMBRYONIC INFECTIONS

The placenta has many functions that support fetal development, including gas exchange, nutrient absorption, fluid regulation, and waste elimination. Transfer of substances across the maternal-fetal barrier depends on factors including its thickness and surface area, the concentration gradient, and the presence of active transport mechanisms (8). With its unique histological architecture, the placenta acts as a specialized immunological and physical barrier and a tightly regulated maternal-fetal interface, protecting the fetus from harmful molecules and invading pathogens (9). However, despite these defenses, the placenta remains susceptible to certain pathogens.

Across different host species, placental structure varies, and the type of placentation is classified based on morphology, cellular organization, and histological structure. In cattle and small ruminants, cotyledonary placentation involves the aggregation of chorionic villi into cotyledons that align with maternal caruncles to form placentomes. These species exhibit a synepitheliochorial placenta, characterized by direct contact between fetal trophoblasts and maternal connective tissue (though early gestation shows an epitheliochorial structure). In contrast, primates and rodents have a discoidal, hemochorial placenta, where trophoblastic invasion erodes maternal tissue, allowing direct contact between the chorion and maternal blood (10).

Structural differences between synepitheliochorial and hemochorial placentas have significant implications for maternal-fetal exchange. The synepitheliochorial placenta establishes a relatively thick barrier between maternal and fetal blood supplies, which limits passive diffusion and requires active or facilitated transport mechanisms to ensure efficient nutrient and gas exchange. Conversely, the hemochorial placenta allows direct contact between the chorion and maternal blood, forming a markedly thinner barrier that significantly enhances permeability. This facilitates rapid and efficient transfer of gases, nutrients, and immunoglobulins but also increases fetal exposure to maternal infectious agents (11).

Several hypotheses have been proposed to explain how pathogens breach the maternal-fetal barrier to infect the developing fetus. Vertical transmission, defined as the passage of an infectious agent from the maternal host to the fetus, represents a major cause of morbidity and mortality during pregnancy. The most widely recognized pathway is hematogenous (transplacental) transmission, in which pathogens circulating in the maternal bloodstream cross the placenta either by directly infecting trophoblasts or by disrupting placental integrity (12). This mechanism has been well documented in a variety of pathogens, including *T. gondii* (13–15), *Brucella* spp. (16–18), and *L. monocytogenes* (19, 20). However, despite these observations, the underlying cellular and molecular mechanisms that govern placental crossing and fetal invasion remain incompletely understood, and the available literature addressing these processes is still limited, particularly when considering differences among host species and placental architectures.

The human placenta is composed of distinct trophoblast subpopulations with specialized functions. After implantation, cytotrophoblasts (CTs) proliferate and differentiate into either syncytiotrophoblasts (STs) or extravillous trophoblasts (EVTs). STs line the placental villi, mediate maternal-fetal exchange, and constitute a major barrier to congenital pathogens, whereas EVTs invade the maternal decidua and remodel spiral arteries to establish uteroplacental circulation (21). Evidence indicates that STs are highly resistant to pathogens, limiting direct invasion from maternal blood (22). CTs also exhibit intrinsic innate immune defenses (22). Consequently, successful transmission requires pathogen-specific strategies to overcome trophoblast-intrinsic barriers, including exploitation of EVTs, dissemination via infected maternal immune cells, or induction of placental inflammation and tissue damage (23).

Placental morphology and function undergo profound changes throughout gestation, including thinning of the trophoblast barrier, increased villous surface area, enhanced maternal blood flow, and dynamic shifts in trophoblast subpopulations and immune regulation (24). While these adaptations are essential to support fetal growth, they may also influence susceptibility to vertical transmission. Progressive reduction in the thickness of the maternal-fetal interface and increased exposure of trophoblasts to maternal blood may facilitate pathogen access to placental tissues, particularly for intracellular pathogens that rely on maternal bacteremia or parasitemia (23).

**Fig 1:**
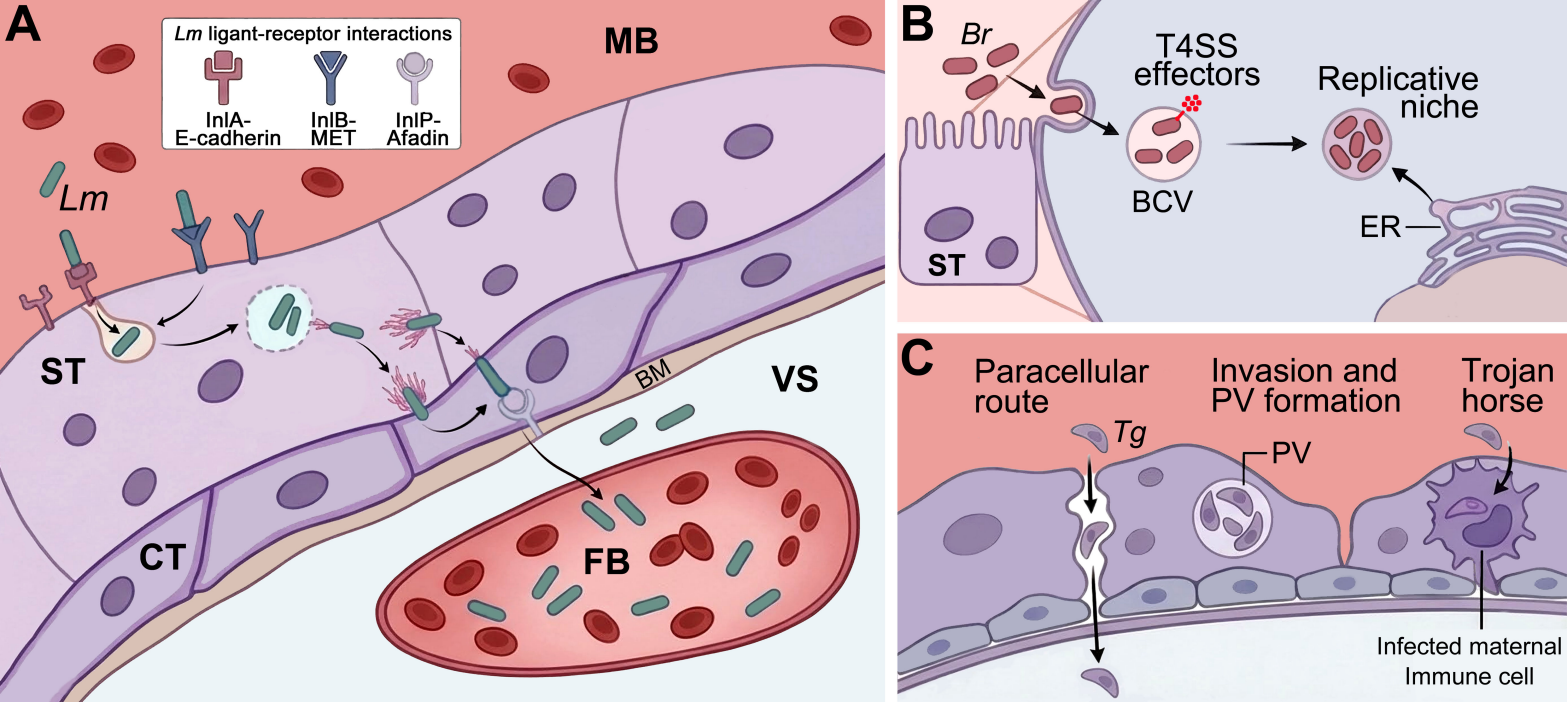
Invasion of placental fetal tissues by zoonotic pathogens. Schematic representation of strategies used by pathogens to cross the human placental barrier and access fetal circulation. (**A**) *Listeria monocytogenes* (*Lm*) reaches the placenta from the maternal bloodstream (MB), inducing receptor-mediated transcytosis through the interaction of host E-cadherin or Met receptors with its surface proteins internalin A (InlA) and B (InlB), respectively. Initially, *L. monocytogenes* is localized in a membrane-bound vacuole, but it escapes the vacuole through the action of listeriolysin O (LLO) and then spreads to other cells utilizing an ActA-elicited actin tail; internalin P (InlP) binds Afadin to cross the cytotrophoblasts (CT) toward the maternal-fetal interface and fetal bloodstream (FB). (**B**) *Brucella* sp. (*Br*) invades syncytiotrophoblasts (ST), forming *Brucella*-containing vacuoles (BCV) where the *Brucella* type IV secretion systems (T4SS) are expressed, translocating its effectors and driving intracellular trafficking toward the *Brucella* replicative niche that incorporates endoplasmic reticulum (ER) markers. (**C**) *Toxoplasma gondii* (*Tg*) tachyzoites may reach fetal tissues through three routes: paracellular translocation, active invasion of host cells to establish a protective parasitophorous vacuole (PV), and a “Trojan horse” strategy involving infected maternal immune cells.

In the context of transplacental infection, *L. monocytogenes*, *Brucella* spp., and *T. gondii* are important examples of pathogens that have evolved strategies to bypass placental barriers (Fig. 1). *Listeria* invades trophoblasts and spreads cell-to-cell to avoid immune detection (25, 26). *T. gondii* infects placental cells, especially trophoblasts, exploiting immune-regulated niches to facilitate vertical transmission (27). Similarly, *Brucella* species, especially *Brucella abortus* and *Brucella melitensis*, exhibit a strong tropism for the placenta, localizing within trophoblastic cells and replicating inside endoplasmic reticulum (ER)-derived vacuoles (28–30).

Zoonotic pathogens can affect various stages of gestation, with the timing of infection playing a critical role in determining outcomes. The embryo and fetus are particularly susceptible to infectious insults owing to the immaturity of their immune systems, the immunological tolerance required for maternal-fetal coexistence, and the intricate structure of the maternal-fetal interface. These vulnerabilities can be exploited by pathogens, leading to embryonic or fetal death, intrauterine growth restriction, placental inflammation, or congenital malformations (31). Embryonic death refers to the loss of the conceptus during the early stages of gestation, often resulting in resorption or early pregnancy loss. Fetal death, which occurs later in gestation after the embryo has developed into a distinct fetus, may manifest as abortion or stillbirth, depending on the species and stage of pregnancy (3). Following intrauterine fetal death, different outcomes may occur, including fetal maceration, fetal mummification, or, more commonly, abortion (Table 1) (32).

**TABLE 1:** Comparative summary of lesions and reproductive outcomes associated with *Listeria monocytogenes*, *Brucella* spp., and *Toxoplasma gondii* according to the developmental stage

Developmental stage	*Listeria monocytogenes*	*Brucella* spp.	*Toxoplasma gondii*
Embryonic stage	Early embryonic death associated with maternal bacteremia and early placental colonization. Embryonic resorption or early pregnancy loss.	Early embryonic loss uncommon. Occasional embryonic death associated with maternal systemic infection.	Embryonic death, resorption, or failure of implantation.
Fetal stage	Abortion or stillbirth, mainly in mid to late gestation. Disseminated fetal infection with hepatic, pulmonary, splenic, and occasionally cerebral microabscesses.	Fetal autolysis, abortion, or stillbirth, typically in late gestation. Suppurative hepatitis, bronchointerstitial pneumonia, and lymphadenitis.	Abortion, stillbirth, or congenital infection (severity inversely related to gestational age). Disseminated fetal lesions, including encephalitis, myocarditis, pneumonia, and hepatic necrosis.
Neonatal period	Early-onset neonatal sepsis and respiratory distress. Late-onset meningitis. High neonatal morbidity and mortality.	Weak or stillborn neonates. Neonatal septicemia and pneumonia. Increased perinatal mortality.	Congenital toxoplasmosis with hydrocephalus, retinochoroiditis, and encephalitis. Survivors may develop chronic neurological or ocular sequelae.

Understanding these stage-specific effects of zoonotic pathogens is essential for elucidating the mechanisms of fetal and neonatal demise and for guiding the development of targeted prevention strategies.

## LISTERIA MONOCYTOGENES

*Listeria monocytogenes* is a gram-positive, facultative intracellular bacterium that is a major cause of foodborne disease. In healthy individuals, it typically causes a non-invasive, self-limiting gastrointestinal infection. However, in high-risk groups including pregnant women, neonates, the elderly, and immunocompromised individuals, the bacterium can cross the intestinal barrier, spread hematogenously, and lead to invasive listeriosis, characterized by severe outcomes like septicemia and meningitis (33). In animals, particularly ruminants, listeriosis commonly manifests as rhombencephalitis, abortion, or septicemia (34). In addition to *L. monocytogenes*, *Listeria ivanovii* is also recognized as an important animal pathogen predominantly affecting ruminants. Although less frequently reported, *L. ivanovii* has a tropism for the reproductive tract and placenta and has been implicated in cases of abortion, stillbirth, and neonatal septicemia in cattle and small ruminants (35, 36). This review focused primarily on *L. monocytogenes*, given its major relevance to human disease and the more extensive literature describing its placental tropism, mechanisms of transmission, and fetal and neonatal pathology across various host species.

A defining feature of *L. monocytogenes* as a fetal pathogen is its ability to disseminate hematogenously following intestinal translocation and to efficiently cross the placental barrier. Although infection may occur at any stage of gestation, adverse reproductive outcomes are most commonly observed during mid-to-late gestation in both humans and ruminants. Gestational timing strongly influences disease outcome: early gestational infection is frequently associated with embryonic loss, whereas later infection more often results in abortion, stillbirth, or severe neonatal disease (4).

*Listeria monocytogenes* invasion of host cells requires expression of surface internalins that mediate receptor-dependent host cell entry. Internalin A (InlA) binds to E-cadherin on epithelial and trophoblastic cells, facilitating bacterial translocation across intestinal and placental barriers (37, 38). In parallel, Internalin B interacts with the host Met receptor tyrosine kinase, activating intracellular signaling pathways that promote bacterial uptake into non-phagocytic cells. Engagement of the Met receptor is particularly important for bacterial dissemination and contributes to the broad cell tropism of *L. monocytogenes*, including cells at the maternal-fetal interface. These receptor-mediated invasion mechanisms are central to placental tropism and vertical transmission (39, 40). Internalin P (InlP) is recognized as a major virulence factor in listeriosis, maternal-fetal *in vivo* and *in vitro* models. InlP enhances *L. monocytogenes* transplacental spread by binding to the host protein Afadin, disrupting trophoblast cell–cell junctions, and facilitating bacterial passage from maternal circulation into fetal tissues (25, 41).

Transplacental transmission following maternal bacteremia represents the primary route of fetal infection. In humans, placental lesions are characterized by acute chorioamnionitis, placentitis, and microabscess, often accompanied by rapid fetal compromise. Fetal pathology reflects systemic hematogenous dissemination and commonly includes multifocal microabscesses in the liver, lungs, spleen, and occasionally the brain (42–45). Similar lesions are observed in ruminants, where infection typically results in necrotizing placentitis involving cotyledonary villi and multifocal necrosis in fetal organs (34, 46). Despite marked differences in placental architecture between humans and ruminants, the pathological hallmark across species is placental inflammation and necrosis leading to impaired maternal-fetal exchange.

Neonatal listeriosis represents the most frequently recognized clinical manifestation of human listeriosis. Early-onset disease is typically characterized by septicemia, respiratory distress, and high mortality rates, particularly among premature neonates. In contrast, late-onset disease, more commonly associated with exposure during or shortly after birth, predominantly presents as meningitis (47, 48).

In human neonates, early-onset listeriosis usually occurs within the first 72 h of life and is associated with sepsis, respiratory distress, fever or hypothermia, hepatosplenomegaly, and, in rare cases, “granulomatosis infantiseptica,” a condition characterized by disseminated necrotizing granulomas in organs such as the liver, spleen, lungs, and skin. Late-onset listeriosis typically appears between 7 and 28 days of life and most commonly presents as meningitis, with clinical signs including fever, lethargy, seizures, and a bulging fontanelle. Lesions in affected neonates may include microabscesses and granulomas in the liver and spleen, interstitial pneumonia in the lungs, and suppurative meningitis or ventriculitis in the brain (49, 50).

Listeriosis in pregnant ewes predominantly results in abortion, stillbirth, or the birth of weak lambs, with outcomes strongly influenced by gestational stage. Early infections are typically associated with placental necrosis and fetal death, whereas late-gestation infections more often lead to full-term stillbirths or the birth of moribund neonates, some of which may survive. The bacterium exhibits a marked tropism for the gravid uterus, crossing the placenta and infecting the fetus via the umbilical circulation, while most ewes recover with minimal or no clinical illness (51, 52).

**Fig 2:**
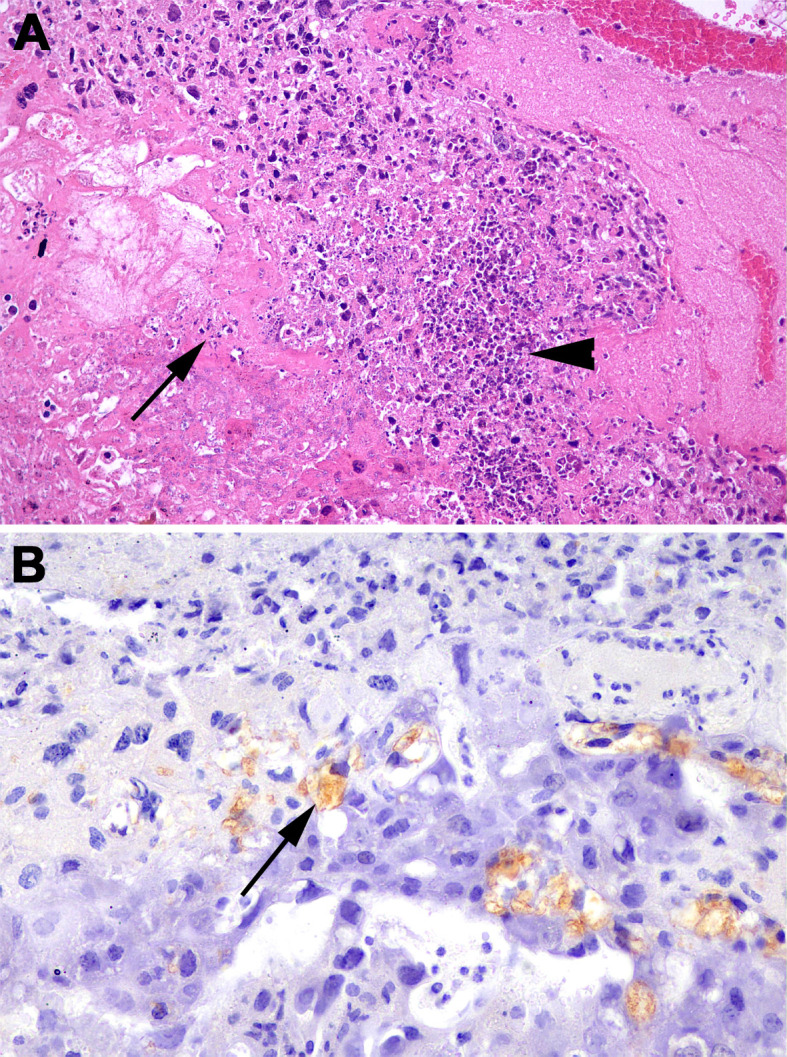
Murine placenta from a maternal-fetal model of *Listeria monocytogenes* infection. (**A**) Extensive areas of necrosis with hypereosinophilic cellular debris and cellular debris (arrow) with marked neutrophilic infiltrate (arrow head). Hematoxylin and eosin, 20× objective. (**B**) Immunolabeling of intralesional *Listeria monocytogenes* (arrow) in the placenta. Immunohistochemistry, 40× objective.

To elucidate the mechanisms of vertical transmission and placental pathology, several maternal-fetal models have been developed. Murine models have been particularly informative, demonstrating that *L. monocytogenes *preferentially targets endovascular trophoblasts, spreading through the spongiotrophoblast and into the labyrinthine zone. Infection is associated with neutrophilic infiltration, microabscesses, and thrombosis, leading to placental necrosis and ischemia (Fig. 2). Fetal infection occurs only when placental colonization is substantial, which emphasizes the importance of bacterial load in vertical transmission (53). Interestingly, even in the absence of detectable intrauterine infection, orally inoculated pregnant mice exhibited reduced fetal size and increased rates of fetal resorption (54). Systemic inflammation alone is sufficient to adversely affect fetal development (55). In the oral infection mice model, the incompatibility between bacterial InlA and mouse E-cadherin restricts intestinal translocation (56), resulting in limited systemic infection and low bacteremia, which is insufficient to colonize the placenta.

Barber et al. (57) demonstrated, in a murine model of placental listeriosis, that Th1-type cytokines such as tumor necrosis factor alpha (TNF-α) and IFN-γ contribute to local immune responses against *L. monocytogenes* at the maternal-fetal interface. In this experimental setting, these cytokines were associated with delayed placental colonization even in the absence of T-cell recruitment, suggesting that cytokine-mediated mechanisms may participate in placental defense. However, these observations derive primarily from experimental models, and the extent to which similar cytokine-driven responses operate in human or natural animal infections remains incompletely understood.

The guinea pig has also served as a model for listeriosis. Following maternal infection, the bacteria proliferated rapidly in the placenta (by as much as 1,000-fold within 24 h). In this model, mutants lacking ActA (actin assembly-inducing protein A) can replicate in the placenta but fail to efficiently invade fetal tissues (58). ActA is an important factor required for cell-to-cell spread, a process by which the bacterium moves directly between adjacent host cells by hijacking the host actin cytoskeleton. This mechanism enables *L. monocytogenes* to evade immune defenses and disseminate effectively through tissues such as the placenta (59).

Non-human primate models, which closely resemble human placentation, confirm the strong tropism of *L. monocytogenes* for the maternal-fetal interface and demonstrate that placental vascular injury and inflammation can lead to acute fetal demise during early gestation (60).

In conclusion, *L. monocytogenes* infection during pregnancy poses a significant risk to fetal and neonatal health due to its ability to cross the placental barrier and invade trophoblasts. Placental colonization leads to inflammation, necrosis, and disruption of maternal-fetal exchange, resulting in spontaneous abortion, stillbirth, or neonatal sepsis. *Listeria* intracellular lifestyle enables it to evade host immune defenses while spreading from maternal tissues to the placenta and fetus. Notably, several of these pathogenic mechanisms, including trophoblast targeting, gestational timing effects, and placental damage, are not unique to *Listeria* but are also observed in infections caused by *Brucella* spp. and *T. gondii*. Comparative evaluation of these agents therefore offers valuable insights into shared and divergent mechanisms driving zoonotic fetal disease.

## *BRUCELLA* SPP.

Brucellosis is a zoonotic disease characterized by reproductive disorders, especially abortion in livestock, and is associated with significant economic losses (61). Humans are typically infected by consuming unpasteurized dairy products or via direct contact with infected animals, placental tissues, or aborted fetuses (62). The genus *Brucella* comprises several classical species, including *B. melitensis*, *B. abortus*, *Brucella suis*, *Brucella canis*, *Brucella ovis*, and *Brucella neotomae*. Over recent decades, taxonomic diversity of the genus *Brucella* has expanded, driven by advances in molecular and genomic approaches and increased investigation of wildlife reservoirs (63).

The clinical course of brucellosis in humans is typically characterized by high, intermittent fever and may progress to a chronic form involving arthritis, hepatitis, orchitis, encephalomyelitis, or endocarditis (64). In contrast to *L. monocytogenes* and *T. gondii*, which represent an important cause of severe gestational and neonatal disease in humans, during pregnancy, brucellosis is associated with less severe reproductive complications. However, adverse obstetric outcomes occur significantly more frequently than in uninfected pregnant women. The most common and severe complications include spontaneous abortion (2.5%–54.5%), intrauterine fetal death (0%–20.6%), and preterm birth (1.2%–28.6%), depending on the gestational stage at infection. Additional adverse outcomes may affect the neonate, including low birth weight, developmental delay, or perinatal death. Prompt diagnosis and initiation of appropriate antimicrobial therapy in pregnant women are essential, as early treatment markedly reduces the likelihood of adverse maternal and fetal outcomes (65).

Reproductive losses in animals are frequent and typically occur during late gestation. While this gestational stage is also when *L. monocytogenes*-elicited abortions are more common, it is important to note that *L. monocytogenes* can induce reproductive failure at various stages of pregnancy (66). In the case of *Brucella* infection, this susceptibility has been attributed, at least in part, to the presence of erythritol and other polyols that may favor bacterial replication in trophoblastic cells, although this mechanism remains incompletely understood (67).

**Fig 3:**
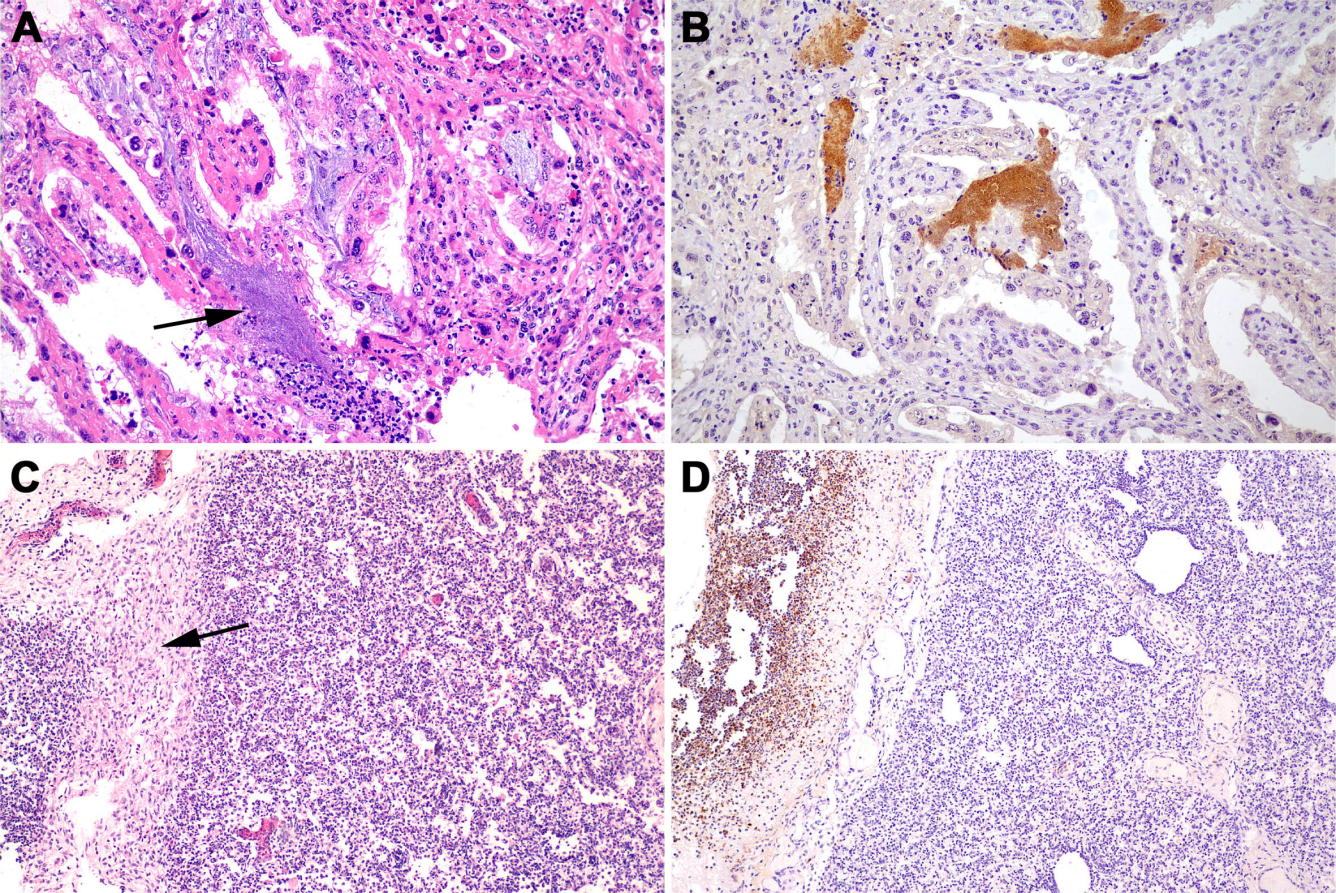
Placenta and fetal lung from a pregnant cow and fetus infected with *Brucella abortus*. (**A**) Neutrophilic and necrotizing placentitis with large intralesional colonies of *B. abortus* (arrow). Hematoxylin and eosin, 20× objective. (**B**) Marked multifocal immunolabeling of *Brucella* sp. in the placenta (brown chromogen). Immunohistochemistry, 20× objective. (**C**) Fetal lung with lymphohistioplasmacytic infiltrate in the pleura (arrow). Hematoxylin and eosin, 10× objective. (**D**) Fetal visceral pleura with immunolabeling of *Brucella* sp. (brown chromogen). Immunohistochemistry, 10× objective.

*Brucella* spp. invade host cells through mechanisms that involve lipid raft microdomains and the formation of a specialized intracellular niche. During entry, *Brucella* preferentially exploits cholesterol-rich lipid rafts in the host cell membrane, which facilitate bacterial uptake by professional and non-professional phagocytic cells. Following internalization, *Brucella* resides within a *Brucella*-containing vacuole (BCV) that initially displays features of the endocytic pathway. However, instead of undergoing lysosomal degradation, the BCV is actively remodeled through the action of the VirB type IV secretion system. VirB-translocated effector proteins modulate host vesicular trafficking, allowing the BCV to evade lysosomal fusion and progressively interact with the endoplasmic reticulum, while it acquires ER-derived membranes (68). The primary pathological feature in reproductive brucellosis is placentitis (Fig. 3), characterized by necrosis of trophoblastic epithelium, vasculitis, and infiltration of macrophages and neutrophils, resulting in impaired maternal-fetal exchange and fetal hypoxia. Dissemination of bacteria to fetal tissues leads to hepatomegaly, splenomegaly, lymphadenitis, interstitial pneumonia, fibrinous pleuritis, pericarditis, or peritonitis. In stillborn and neonatal cases, lesions often involve suppurative bronchopneumonia, hepatic necrosis, and meningoencephalitis, reflecting systemic infection and septicemia (69–71).

Microscopically, fetal infection with *Brucella* spp. is characterized by necrosuppurative or interstitial pneumonia, inflammatory infiltrates composed of neutrophils, lymphocytes, and macrophages, and frequent involvement of serosal surfaces, often accompanied by placentitis (Fig. 3). Additional lesions may include hepatocellular necrosis and, less consistently, involvement of the liver, kidney, and central nervous system, reflecting systemic fetal infection (70–73).

Experimental and natural infections across animal species, including goats (73), pigs (74), and dogs (75, 76), provide consistent evidence of vertical transmission and progressive fetal involvement in brucellosis. Infected dams frequently produce aborted or stillborn fetuses, particularly following prolonged infection, and *Brucella* antigens are widely distributed in placental and fetal tissues. Lesions are dominated by necrotizing placentitis and systemic fetal infection, with bacterial localization primarily in trophoblasts and macrophages, although broader cell tropism has been described (75). Although different *Brucella* species share common pathogenic features during pregnancy, the patterns and severity of reproductive and fetal pathology vary according to the bacterial species and its natural host. *B. abortus* and *B. melitensis*, which are highly adapted to ruminants, typically induce severe necrosuppurative placentitis and late-gestation abortion (72, 73). In contrast, *B. suis* infection in pigs is often associated with necro-hemorrhagic placentitis and widespread fetal septicemic lesions (74), whereas *B. canis* infection in dogs tends to produce more disseminated fetal infection (75), although it is also associated with placental inflammation (76).

The pregnant murine model has been widely used to investigate the mechanisms of *Brucella* infection during gestation and provides a valuable model for studying the intracellular replication of *B. abortus*, *B. melitensis*, and *B. ovis* within trophoblasts (77–79). Experimental infection demonstrates that *Brucella* spp. replicate intracellularly in trophoblasts, where bacteria localize in the rough endoplasmic reticulum of giant trophoblastic cells, inducing necrosuppurative placentitis. Inflammatory infiltrates, including neutrophils within the decidua and uterine lumen, are commonly observed. In severely affected placentas, infarction of the labyrinthine zone may occur, ultimately leading to fetal death (77). Similar placental pathology, characterized by neutrophilic necrotizing placentitis, has also been reported following infection with *B. ovis* in pregnant mice (79).

In a pregnant mouse model, *B. melitensis* infection (78) resulted in dose-dependent adverse pregnancy outcomes, with stillbirths occurring at higher inoculum levels despite the absence of overt abortion. Placentas associated with stillborn fetuses were reduced in weight and showed bacterial localization within giant trophoblastic cells, indicating efficient vertical transmission. Systemic maternal involvement, including splenomegaly and uterine hemorrhage, accompanied high-dose infection, with bacterial burdens in the placenta exceeding those in fetal or splenic tissues.

Although the pathogenesis of abortion in brucellosis is not fully understood, experimental studies indicate a key role for interferon-gamma (IFN-γ). In pregnant mice infected with *B. abortus*, blockade of IFN-γ signaling prevented abortion, demonstrating that IFN-γ-mediated immune responses contribute directly to fetal loss (80). Studies on the pathogenesis have also demonstrated that ER stress and activation of the unfolded protein response play central roles in *Brucella*-associated placental pathology. During *B. abortus* infection, the type IV secretion system induces ER stress in trophoblasts via the effector VceC (VirB co-regulated effector C), leading to CHOP (C/EBP homologous protein)-dependent trophoblast cell death, placental inflammation, and fetal loss (81). ER stress-driven increases in systemic TNF-α further exacerbate placental damage, while TNF-α blockade mitigates inflammation and improves fetal viability in experimental models *(82*).

In summary, infection with *Brucella* species during pregnancy can lead to significant fetal and neonatal outcomes, primarily through placental colonization, trophoblast infection, and induction of inflammatory responses. Similar to *Listeria monocytogenes*, *Brucella* exhibits a marked tropism for placental tissues, leading to disruption of placental architecture, necrosis, vascular injury, and impaired maternal-fetal exchange. However, in contrast to *Listeria*, which often causes acute systemic maternal infection with rapid hematogenous placental invasion, *Brucella* is characterized by chronic intracellular persistence and a more insidious progression of placental disease. Mechanistically, in the case of *Brucella* infection, the interplay between bacterial intracellular survival, induction of endoplasmic reticulum stress, and host immune responses, including cytokine-mediated inflammation, contributes to placental dysfunction and fetal demise.

## TOXOPLASMA GONDII

*Toxoplasma gondii* is a widespread apicomplexan protozoan of considerable significance in human and veterinary medicine. As an obligate intracellular parasite, *T. gondii* has the sexual stage of its life cycle in cats and wild felids, the definitive hosts, while the asexual phase occurs in a wide variety of warm-blooded intermediate hosts, including rodents, sheep, goats, pigs, poultry, and humans. Transmission typically occurs through the ingestion of oocysts shed in feces of the definitive hosts or tissue cysts present in undercooked meat from infected intermediate hosts. The parasite is capable of crossing the blood-brain, blood-retinal, and placental barriers, facilitating vertical transmission from mother to fetus (19).

Sheep are highly susceptible to reproductive toxoplasmosis, with primary infection during pregnancy frequently resulting in abortion and significant economic losses (83). The disease is characterized by placental invasion, necrotizing placentitis, and fetal lesions involving organs such as the liver and brain (84). Importantly, gestational age at the time of infection is a major determinant of outcome: early infections, when fetal immune competence is limited, lead to severe placental and fetal damage, abortion, or mummification, whereas later infections more often result in the birth of live but congenitally infected lambs (85). In ovine toxoplasmosis, sterile abortion (fetal loss in the absence of detectable *T. gondii* in fetal tissues) is a well-recognized clinical outcome. This phenomenon is attributed to severe placentitis and placental dysfunction that compromise fetal viability even without direct fetal infection (86).

Local and systemic immune responses play a central role in the pathogenesis of toxoplasmosis. Increased placental production of IFN-γ and TNF-α, along with variable expression of interleukin-4 and interleukin-10, reflects the delicate immunological balance between parasite control and tissue injury. During the acute phase of infection, a pronounced upregulation of Th1-type cytokines, especially IFN-γ and TNF-α, is evident in both maternal circulation and placental tissues (87, 88). These cytokines are critical for controlling tachyzoite replication, with IFN-γ activating macrophages and stimulating nitric oxide production, and TNF-α enhancing parasite clearance. However, excessive or prolonged Th1-type responses can damage placental tissues by inducing apoptosis and compromising vascular integrity, thereby contributing to necrotizing placentitis and fetal hypoxia (87).

Although serological surveys frequently detect antibodies to *T. gondii* in cattle, clinical disease and abortion are rare in this species (89, 90). Molecular investigations have occasionally detected *T. gondii* DNA in aborted bovine fetuses (91). However, other studies found no *T. gondii* DNA in any bovine fetal samples examined (92), and experimental infections in cows have failed to reproduce fetal lesions (93).

In humans, acute *T. gondii* infection during pregnancy is relatively uncommon but can result in transplacental transmission and fetal infection (94). The risk and severity of congenital disease are strongly influenced by gestational age at maternal infection, parasite burden, and parasite virulence. Early gestational infections are less frequently transmitted but are associated with severe fetal damage or death, whereas infections acquired later in pregnancy show higher transmission rates with generally milder or subclinical outcomes (94).

Maternal infection with *T. gondii* is often asymptomatic but may lead to placental infection and subsequent fetal involvement. Fetal infection is initially systemic and may later become localized to the central nervous system. Congenital toxoplasmosis presents a broad clinical spectrum, with predominant neurological manifestations including hydrocephalus, intracranial calcifications, cerebral atrophy, and neurodevelopmental impairment (94–96). Prenatal imaging commonly reveals ventriculomegaly, parenchymal volume loss, and calcifications (97). These lesions result from a combination of direct parasitic damage and indirect effects of placental inflammation, insufficiency, and fetal hypoxia. Ocular involvement is also a prominent manifestation of congenital toxoplasmosis, most frequently manifesting as chorioretinitis and retinochoroidal necrosis. Histopathological changes include disruption of the retinal pigment epithelium and choroidal inflammation, which may progress to chorioretinal scarring and long-term visual impairment. Less commonly, developmental ocular anomalies such as microphthalmia or cataracts may occur (98, 99).

Experimental models have been fundamental in elucidating the mechanisms of congenital toxoplasmosis. Murine studies demonstrate that vertical transmission of *T. gondii* is dose dependent and occurs predominantly following primary maternal infection during pregnancy, whereas prior immunity effectively prevents fetal infection. Parasite burdens are typically highest in maternal tissues and placenta, highlighting the placenta as a key barrier and target (100).

Murine models of congenital toxoplasmosis have shown that vertical transmission occurs mainly when mice are infected for the first time during pregnancy, whereas dams with prior exposure or infection before mating do not transmit the parasite to their offspring, indicating that maternal immunity effectively prevents congenital infection. Approximately half of the pups born to first-time infected dams became infected, while maternal tissue cyst burdens did not correlate with transmission. These findings demonstrate the importance of maternal immune status and infection timing in determining the risk of congenital toxoplasmosis, reflecting human observations where primary maternal infection during pregnancy is the main risk factor for fetal infection and adverse outcomes (101, 102).

Among murine strains, C57BL/6 mice exhibit higher susceptibility due to strong Th1-type immune responses, characterized by elevated IFN-γ and TNF-α levels, which can adversely affect embryonic development even at low infection doses. Although useful for studying maternal immune responses, severe maternal disease in this model may limit its utility for dissecting fetal-specific effects compared with more resistant strains such as BALB/c mice (103).

Rats have been used as experimental models for congenital toxoplasmosis due to their larger size and suitability for placental and fetal analyses. In this model, maternal infection induces alterations in trophoblast populations, placental architecture, and fetal development, with parasite replication and vertical transmission influenced by the kinetics and timing of maternal infection (104). Ferro et al. (105) using the South American rodent *Calomys callosus* demonstrated that maternal infection occurring shortly before or during gestation results in more pronounced disruption of trophoblast layers compared with infections at later stages of gestation, suggesting that time of infection relative to placental development critically shapes placental integrity and contributes to adverse fetal outcomes, even when direct fetal parasitism is limited.

A study by Vargas-Villavicencio et al. (106) used pregnant BALB/c mice to investigate congenital toxoplasmosis and found that some fetuses were resorbed, aborted, or exhibited tissue damage despite the absence of detectable parasites, a phenomenon referred to as “sterile” fetal injury. This indicates that fetal compromise can occur independently of direct parasite transmission. In their murine model using the non-virulent ME49 strain, maternal spleens contained substantially higher parasite loads than placentas, and the placenta retained most of the parasites, effectively limiting vertical transmission. Nevertheless, placental inflammation, necrosis, and vascular alterations were sufficient to impair fetal development, leading to morphological and histopathological abnormalities. These findings demonstrate that congenital toxoplasmosis can affect fetal outcomes through both limited vertical transmission and indirect effects mediated by placental dysfunction or maternal immune responses.

In conclusion, *T. gondii* is an important agent in fetal and neonatal mortality associated with zoonotic infections, primarily through its ability to invade and replicate within placental and fetal tissues. The pathogen induces a spectrum of lesions, including necrosis and inflammation in various fetal organs, which compromise fetal development and viability. In *T. gondii* infection, the timing of maternal exposure critically influences both fetal disease severity and the likelihood of vertical transmission. Early gestational infections are less frequently transmitted but cause more severe placental and fetal lesions, whereas later infections are transmitted more efficiently and generally result in milder disease. This inverse relationship between transmission efficiency and disease severity is characteristic of congenital toxoplasmosis. In contrast, *Listeria monocytogenes* and *Brucella* spp. infections are more consistently associated with adverse fetal outcomes at late gestation. A comprehensive understanding of these mechanisms is essential for designing effective preventive, diagnostic, and therapeutic strategies to reduce reproductive losses in both humans and animals exposed to this zoonotic pathogen.

## CONCLUDING REMARKS

Zoonotic infections caused by *L. monocytogenes*, *Brucella* spp., and *T. gondii* remain major challenges for public health and livestock production. Despite their distinct biological characteristics, these pathogens share the ability to breach placental defenses and establish intracellular niches that disrupt placental integrity and fetal development.

Clinical outcomes are strongly influenced by the gestational stage at infection. Early infections are commonly associated with embryonic loss and resorption, whereas later infections more frequently result in necrotizing placentitis, abortion, stillbirth, and severe neonatal disease. These outcomes are driven by pathogen-specific yet convergent mechanisms, including *Brucella*-induced endoplasmic reticulum stress, *Listeria* cell-to-cell dissemination, and *T. gondii* invasion of trophoblasts coupled with immune-mediated placental injury driven by Th1-type cytokine responses, all of which facilitate immune evasion and placental dysfunction at the maternal-fetal interface.

Although experimental models have advanced our understanding of vertical transmission, important gaps remain in the systematic characterization of fetal and neonatal pathology. Strengthening integrated histopathological and molecular approaches is therefore essential. Improved insight into host-pathogen interactions at the maternal-fetal interface will enhance prevention and management strategies and help mitigate the socioeconomic and sanitary impacts of zoonotic reproductive diseases within a One Health framework.
